# Clinical Characteristics and Whole Exome Sequencing Analysis in Serbian Cases of Clubfoot Deformity—Single Center Study

**DOI:** 10.3390/children11060647

**Published:** 2024-05-27

**Authors:** Filip Milanovic, Sinisa Ducic, Milena Jankovic, Sanja Sindjic-Antunovic, Emilija Dubljanin-Raspopović, Milica Aleksic, Goran Djuricic, Dejan Nikolic

**Affiliations:** 1 Pediatric Surgery Department, University Children’s Hospital, 11000 Belgrade, Serbia; direktor@udk.bg.ac.rs (S.D.); sanja.sindjic@udk.bg.ac.rs (S.S.-A.); 2 Faculty of Medicine, University of Belgrade, 11000 Belgrade, Serbia; milena.jankovic.82@gmail.com (M.J.); emilija.dubljanin-raspopovic@med.bg.ac.rs (E.D.-R.); aleksic.milica@yahoo.com (M.A.); goran.djuricic@udk.bg.ac.rs (G.D.); dejan.nikolic@udk.bg.ac.rs (D.N.); 3 Neurology Clinic, University Clinical Center of Serbia, 11000 Belgrade, Serbia; 4 Center for Physical Medicine and Rehabilitation, University Clinical Center of Serbia, 11000 Belgrade, Serbia; 5 Radiology Department, University Children’s Hospital, 11000 Belgrade, Serbia; 6 Department of Physical Medicine and Rehabilitation, University Children’s Hospital, 11000 Belgrade, Serbia

**Keywords:** clubfoot, whole exome sequencing, TRPV4, FLNB, FOXP1, CNV

## Abstract

Background: Recognized as one of the most serious musculoskeletal deformities, occurring in 1–2 per 1000 newborns, 80% of clubfeet are idiopathic while 20% present with associated malformations. The etiopathogenesis of clubfoot is described as multifactorial, including both genetic and environmental risk factors. The aim of this study was to analyze possible genetic causes of isolated and syndromic clubfoot in Serbian children, as well as to correlate clinical and genetic characteristics that would provide insight into clubfoot etiopathogenesis and possibly contribute to global knowledge about clinical features of different genetically defined disorders. Methods: We evaluated 50 randomly selected, eligible children with clubfoot aged 3 to 16 years that were initially hospitalized and treated at University Children’s Hospital between November 2006 and November 2022. The tested parameters were gender, age, dominant foot, affected foot, degree of deformity, treatment, neuromuscular disorders, positive family history, and maternal smoking. According to the presence of defined genetic mutation/s by whole exome sequencing (WES), patients were separated into two groups: positive (with genetic mutation/s) and negative (without genetic mutation/s). Results: Seven patients were found to be positive, i.e., with genetic mutation/s. A statistically significant difference between categorical variables was found for families with a history of clubfoot, where more than half (57.14%) of patients with confirmed genetic mutation/s also had a family history of genetic mutation/s (*p* = 0.023). Conclusions: The results from this study further expand the genetic epidemiology of clubfoot. This study contributes to the establishment of genetic diagnostic strategies in pediatric patients with this condition, which can lead to more efficient genetic diagnosis.

## 1. Introduction

Recognized as one of the most serious musculoskeletal deformities, clubfoot consists of ankle equinus, hindfoot varus, cavus, forefoot adduction, and calf muscle atrophy [1,2,3,4]. As a part of the congenital dysplasia of all musculoskeletal and neurovascular tissues distal to the knee, such as bone deformities and soft-tissue fibromatosis, clubfoot occurs in around 174,000 children annually, mostly in low-income or middle-income countries [4,5,6,7,8]. Although the approximate incidence is 1–2 in 1000 Caucasian children, data from the literature suggest a varying clubfoot occurrence among different ethnic populations [1,8]. For example, rates as high as 7 in 1000 live births are reported in Hawaiian and Māori children as opposed to rates as low as 0.5–1 in 1000 live births in Japanese and Filipino children [3]. Between 30 and 50% of clubfeet are bilateral, while right-sided deformity is more common in unilateral cases [8,9,10,11,12,13,14,15]. Furthermore, the exact cause of clubfoot in males still remains unclear, although some authors point out the Carter effect, which sets out that females require a greater genetic load to manifest the disorder compared to males [10]. Data from the literature have shown that about 80% of clubfeet are idiopathic, while the remaining 20% present with associated malformations, usually as part of a syndromic neurologic disorder, such as arthrogryposis, cerebral palsy, or myelomeningocele [16,17]. Although syndromic clubfeet are approximately four times less common, they are more challenging to treat and have higher recurrence rates [2,3]. Treatments for clubfoot that are considered challenging are Ponseti stretching, casting, and further use of foot abduction braces, while surgical procedures are necessary for cases of rigid deformity which have proven resistant to previous conservative treatment [3,4,5,10,18,19,20].

It is also worth mentioning that the etiopathogenesis of clubfoot, which is the subject of scientific debates worldwide, as a multifactorial model including both complex genetic and environmental risk factors is likely to describe possible causes of both structural and functional deformities that are easily recognizable at birth, although prenatal ultrasonography is also used to make an early diagnosis [3,4,5,10,11]. Many authors believe that genetic factors play an important role in clubfoot etiopathogenesis, as data from the literature point to a genetic compatibility of around 35% in affected monozygotic twins. This is supported by the fact that nearly one quarter of all clubfoot cases are familial [4,10,21]. Thus, different theories have determined that several genes and their polymorphisms play a role in clubfoot etiopathogenesis while other factors might include in utero positioning, abnormal muscle insertions, anterior tibial artery hypoplasia, elevated maternal homocysteine levels during pregnancy, viral infections, peroneal nerve compression, maternal smoking, early amniocentesis due to fluid leakage, and following oligohydramniosis [4,10,17,22,23,24,25,26,27,28,29,30]. Genes, whose encoded products in the form of the contractile proteins of skeletal myofibers that are involved in muscle morphogenesis and limb development, such as the PITX1-TBX4 transcriptional pathway, chromosome 17q23, and HOX, have also been studied, as their mutations are thought to increase the risk for clubfoot appearance in a newborn [3,10,25,31,32]. N-acetylation genes (NAT1, NAT2), whose encoded products have an impact on modulating tobacco smoke, were also studied, along with other xenobiotic metabolism genes (CYP1A) [1,2]. As the clubfoot deformity shows high genetic heterogeneity, it is recommended to implement the high-throughput, next-generation sequencing methods, as whole genome sequencing (WGS) or whole exome sequencing (WES), in genetic studies in different populations.

Therefore, the aim of this study was to analyze possible genetic causes of isolated and syndromic clubfoot in Serbian pediatric patients, in order to provide a better understanding of the genetic epidemiology of this condition in Serbia. Additionally, the study aimed to correlate clinical and genetic characteristics that would provide insight into clubfoot etiopathogenesis and possibly contribute to global knowledge about the clinical features of different genetically defined disorders.

## 2. Methodology

### 2.1. Study Design and Participants

The prospective interventional study included 50 randomly selected patients with a diagnosed clubfoot deformity who were admitted for additional diagnostics and examination at University Children’s Hospital (UCH) in Belgrade, Serbia, between November 2022 and March 2023 (follow-up examination at UCH). Eligible patients were selected from the medical records of the UCH database by the diagnosis of clubfoot when they were admitted for hospitalization and surgical intervention (initial hospitalization at UCH). From the computer database, 50 patients out of 473 children were randomly selected by the method where every fifth patient up to the number of 50 was included. All of the included patients completed the study. There were no dropouts of patients during the study. The period for search in the hospital medical records from the period of hospitalization and surgical intervention was set between November 2006 and November 2022. Clubfoot was diagnosed by the board-certified pediatric orthopedic surgeon or the board-certified pediatric physiatrist. The decision for initial treatment by the Ponseti method was made by the board-certified pediatric orthopedic surgeon. The information regarding the children’s ages (between three and 16 years) was taken at the visit from the follow-up examination at the UCH for study participation. According to the presence of defined genetic mutation/s by whole exome sequencing (WES), patients were selected in two groups: positive (with genetic mutation/s) and negative (without genetic mutation/s).

The primary outcome of the study was genetic evaluation in patients with clubfoot, while secondary outcomes were the evaluation of clubfoot family history, presence of neuromuscular disorders, and treatment modality.

The study was conducted according to the principles of good clinical practice and the Declaration of Helsinki, as well as being approved by the Institutional Review Board of University Children’s Hospital (Number 16/34; Date: 14 December 2022). Prior to inclusion in the study, informed consent was obtained from parents or legal guardians.

### 2.2. Clinical and Demographic Characteristics

Further parameters were tested: gender, age, dominant foot, affected foot, degree of deformity, treatment, neuromuscular disorders, positive family history, and maternal smoking. Gender was defined as male and female. The dominant foot was defined as right or left. Even though the ideal method for leg dominance determination is still lacking [33], previously it was proposed that the preferred leg differs between dynamic and static tasks [34]. Therefore, patients were asked to perform both tasks. For the dynamic task, the patients were asked to kick a ball and for the static task to balance on one leg. The right or left dominance was defined as performing both tasks on the same leg. Previous reports state that children before 3 years of age rely on vision to maintain balance, while in the period between 3 and 6 years, children start to learn how to use and integrate visual, vestibular, and proprioception sensory information to maintain balance [35]; therefore, we included children aged 3 and above.

The affected foot was defined as one foot or both feet. The degree of deformity was categorized as I, II, III, or IV according to Dimeglio classification [1], where degree I is considered as a soft clubfoot, reducible without significant resistance; degree II as moderate clubfoot that is reducible with some resistance; degree III as severe clubfoot that is reducible only against significant resistance; and degree IV as very severe or syndromic clubfoot. The treatment modalities included the Ponseti method or the Ponseti method with extensive surgical treatment [2,3,4,10,31,32]. Neuromuscular disorders were defined depending on their presence (present or absent) [16,17]. A family history of clubfoot was considered as positive if a first- or second-degree relative was affected or negative if there was no one affected in these categories of relatives [36]. Maternal smoking was defined as smoking at least one cigarette a day during pregnancy [37].

### 2.3. Genetic Analysis

The peripheral blood samples of 50 selected patients were collected in vacutainers with EDTA, and genomic DNA was extracted using standard protocols by the 3Billion Company, Seoul, Republic of Korea. To perform the whole exome sequencing (WES), the xGen Exome Research Panel v2, xGen human mtDNA panel, and xGen Custom Hyb Panel v1 (Integrated DNA Technologies, Coralville, IA, USA) were applied. Sequencing was performed on the NovaSeq 6000 platform (Illumina, San Diego, CA, USA).

The generated sequences were aligned to the Genome Reference Consortium Human Build 37 (GRCh37) and Revised Cambridge Reference Sequence (rCRS) of the mitochondrial genome. In all analyzed samples, the sequencing data had a high quality and specific coverage; in addition, the list of single-nucleotide variants (SNVs) and small insertions and deletions (indels) that were identified are available upon request.

EVIDENCE, an automated variant prioritization system, was applied for variant interpretation [38], based on clinical data and relevant family history, combined with ACMG/AMP recommendations [39].

Additionally, several relevant databases were searched in order to characterize variants more precisely and to establish clinical and genetic correlations (OMIM^®^ (Online Mendelian Inheritance in Man) database, http://omim.org/downloads (accessed on 19 February 2024); gnomAD (genome aggregation database), https://gnomad.broadinstitute.org (accessed on 19 February 2024); ClinVar (National Center for Biotechnology Information ClinVar Database), https://ncbi.nlm.nih.gov/clinvar (accessed on 20 February 2024); HGVS (Human Genome Variation Society), https://varnomen.hgvs.org (accessed on 20 February 2024); HGMD (The Human Gene Mutation Database), https://www.hgmd.cf.ac.uk/ac/index.php (accessed on 20 February 2024)).

Segregation analysis was performed in the Laboratory for Molecular and Genetic Diagnostic of Neurological Diseases, Neurology Clinic, University Clinical Center of Serbia, Belgrade, Serbia. The testing of available biological parents and/or other family members to confirm the segregation of the variant included the direct sequencing method (ABI3500, Applied Bioscience) for single-nucleotide variants or QF-PCR (ABI7500 Fast, Applied Bioscience) for copy number variants.

Carriers of pathogenic/likely pathogenic variants, as well as single variants of unknown significance, were marked as positive patients, while no-mutation carriers were marked as negative patients.

### 2.4. Diagnostic Protocol for Clubfoot

As a teaching university hospital for children and one among the few referral centers with experts in the field of pediatric orthopedic surgery and rehabilitation, patients with a suspicion of clubfoot deformity are referred for assessment, diagnostics, and treatment.

On the initial examination, board-certified pediatric orthopedic surgeon or board-certified pediatric physiatrist assessed the range of motion (RoM) and stability of ankle and foot joints in patients with clubfeet. The degree of deformity was then assessed by the Dimeglio classification [1]. In cases where there were doubts regarding the diagnosis, severity, and stability of the presented clubfoot, radiological examinations were performed.

### 2.5. Treatment Protocol for Clubfoot

In all patients with clubfoot from our study, the Ponseti method was performed through 3 phases that included: manipulation and casting (Phase I), tenotomy (Phase II), and bracing (Phase III) [3]. Study participants were included in the treatment between 1 and 3 weeks of life [3].

In cases where satisfactory correction was not achieved after Phase III of Ponseti treatment, extensive surgical procedures were performed for deformity correction. The choice of surgical procedure was made by the board-certified orthopedic surgeon according to the components that should be corrected.

### 2.6. Statistical Analysis

The sample size was calculated by the sample size calculator [40], with regards to the presence of additional anomalies in children with idiopathic congenital equinovarus of the New Zealand European ethnicity group [41]. The sample size was estimated at 44 participants for the confidence level of 80% and margin of error of 5%. We included an additional 6 more patients in case of dropouts.

The results are presented as the mean value (MV) and standard deviation (SD) for continuous variables, while categorical variables are presented as the whole numbers (N) and percentages (%). To evaluate the statistical difference of the categorical variables between the tested groups (positive and negative), we used Fisher’s test. Data were statistically analyzed using IBM SPSS software, version 21 (IBM Corporation, Armonk, NY, USA). Statistical significance was set at *p* < 0.05.

## 3. Results

The distribution of the tested parameters in the study sample is presented in Table 1. The study results show a male predominance of 62%. Right-footed patients accounted for around two-thirds of patients (66%). Both feet were affected in almost three-quarters of patients (74%). For those unilateral cases, nine (18%) patients presented with right-sided and four (8%) patients with left-sided deformity. The most dominant types of the deformity were Types II (34%) and III (28%) according to Dimeglio. The Ponseti method followed by the extensive surgical treatment procedure was performed in 72% of patients, while the Ponseti method only was performed in 28% of patients. Associated neuromuscular disorders were noticed in less than half of patients (42%), while a positive clubfoot family history was found in every fifth patient (20%). Smoking during pregnancy was observed in almost every third mother (32%). No statistically significant difference in the categorical variables between the tested groups was found except for a clubfoot family history where more than half (57.14%) of patients with confirmed genetic mutation/s had a positive family history (*p* = 0.023).

Individual characteristics in the group of seven genetically positive patients are presented in Table 2. Approximately 70% of the patients were males and around 85% of the tested participants were right-footed. All the patients had both their feet affected. There were three (42.86%) patients with Grade II according to Dimeglio classification and four (57.14%) patients with Grade III. Among all patients with confirmed genetic mutation/s and with associated neuromuscular disorder, four (57.14%) had a positive clubfoot family history while positive maternal smoking during pregnancy was confirmed in one (14.29%) case. One patient was treated by the Ponseti method while the remaining six (85.71%) patients were treated by the Ponseti method followed by the extensive surgery intervention.

In total, seven patients were carriers of six different heterozygous variants (Table 3). Five variants were characterized as pathogenic or likely pathogenic, and one variant was of unknown significance.

The two different heterozygous missense variants were identified in the gene *TRPV4*, in a single patient each. One variant (c.806G>A) is a known pathogenic variant, previously associated with hereditary motor and sensory neuropathy, Type IIc, while the pathogenicity of the other variant (c.1315T>A) was not confirmed. Another heterozygous missense variant (p.Gln685His), identified in the gene *FLNB* in monozygotic twins, was characterized as likely pathogenic and associated with Larsen syndrome. In one patient, we found a novel heterozygous *FOXP1* intronic variant (c.1723-2A>C), predicted to affect splicing and also characterized as a likely pathogenic and possible cause of FOXP1-related disorder.

In our cohort, two larger genomic rearrangements were also identified, and both were duplications. One patient has a 17p12 region duplication (minimum size: 1.4 Mb) associated with Charcot–Marie–Tooth disease, Type 1A, and another patient has a duplication of chromosome Xq28 region (minimum size: 435.6 Kb).

For the remaining 43 patients, clinically significant variants that could be relevant to the patients’ phenotypes were not identified.

## 4. Discussion

Our study represents the first comprehensive analysis of the genetic variants associated with clubfoot deformity in the Serbian population and, accordingly, may contribute to a better understanding of the genetic role in heterogeneous clubfoot etiology.

The results of our study demonstrated that the positive family history is significantly more frequent in clubfoot patients with genetic variants. A total of 7 (14%) out of 50 tested subjects were carriers of six different heterozygous variants, and a positive clubfoot family history was found in almost every fifth tested patient. Similar familial occurrences are reported in other studies [3,10,18]. Furthermore, all of the patients with genetic variants had some type of neuromuscular disorder, and the majority of these patients (six out of seven patients) were treated by the Ponseti method followed by extensive surgery.

Our results showed a male predominance of around 1.5:1, as around 60% of patients with clubfoot were males while around 40% were females. Similar findings were previously reported by different studies [3,10], pointing out the Carter effect, in which females require a greater genetic load to manifest the disorder compared to males [42]. Bilateral deformity was present in around three-quarters of patients, while the literature describes that 30–50% of clubfoot cases are bilateral. The right-sided preponderance for unilateral cases described in the literature was also shown in our study, as 9 out of 13 patients had a right-sided deformity [8,9,10,11,12,13,14,15]. Furthermore, we used the Dimeglio classification system—simple, reproducible, and reliable—to describe the severity of the condition; most of the patients (34%) in our study had Type II clubfoot according to Dimeglio, or moderate clubfoot that is reducible with some resistance [43]. Moreover, while the literature [16,17] describes that only 20% of clubfeet are associated with other malformations, usually as part of syndromic neurologic disorders, such as arthrogryposis, cerebral palsy, or myelomeningocele, while the rest are isolated, our research showed a higher incidence of syndromic clubfoot of around 40%, which can be explained both by the small sample and that UCH is considered as a major referral center where complex orthopedic pathology from the entire country is referred and treated. In addition to this, the presence of muscular abnormalities in patients with idiopathic clubfeet is described in the study of Lampasi et al., with the frequency of 31.5% at presentation [44].

Finally, almost every third patient was treated by the Ponseti method only, while the rest were treated by the Ponseti method along with additional surgical procedures. That is not in accordance with other studies [3,45,46,47], describing the Ponseti method as the gold standard, superior for the treatment of clubfoot. This discrepancy could be potentially explained by the fact that the patients who were treated at the UCH were resistant to previous treatment by the Ponseti method only.

As mentioned earlier, a statistically significant difference in the categorical variables was found for a clubfoot family history, where more than half (57.14%) of patients with confirmed genetic mutation/s had a positive family history of clubfoot. This finding correlates with other studies, suggesting that nearly one-quarter of all clubfoot cases are familial [4,10,21].

In the systematic review of Pavone et al., it was shown that several gene families and pathways (HOX family; PITX1-TBX4 pathway; apoptotic pathway; muscle contractile protein—troponin and tropomyosin) contribute to clubfoot development [48]. PITX1 is involved in hindlimb identity; TBX4 is described as a hindlimb-specific T-box transcription factor participating in the muscles’ and tendons’ patterning [49]. Although no major gene in clubfoot etiopathogenesis has been identified, variants in the genes *PITX1* and *TBX4* have been intensively studied, but without consistent results [49,50]. Bianco et al. suggested that high heterogeneity between patients is preventing the identification of the common genetic cause or major gene behind clubfoot [50]. Therefore, WES would be a suitable approach and more widely recommended in the diagnosis of disorders with complex etiologies and a strong genetic component. Our WES results support the genetic heterogeneity of clubfoot. A probable genetic cause of the disorder was identified in 14% of our patients or 12% if we exclude the variant of unknown significance, and *TRPV4* variants are the only ones associated with the condition in more than one unrelated patient. Two different heterozygous variants were identified in coding regions of *TRPV4*, the gene associated with several skeletal and neuromuscular disorders, as well as with digital arthropathy-brachydactyly [51].

The variant p.Arg269His was found in a patient with bilateral clubfoot and numerous additional symptoms: congenital hip dislocations, vocal cord paralysis, arthrogryposis, and motoric delay for age. The parents of our proband were unaffected and targeted parental testing revealed that the variant is absent in both parents and thus confirmed that the variant is de novo. The same variant has previously been reported in the literature as pathogenic/likely pathogenic in several unrelated patients with similar clinical presentation and it co-segregated with the disease in families [52,53,54,55,56]. Additionally, two different pathogenic/likely pathogenic amino acid changes at the same codon have been reported (p.Arg269Cys, p.Arg269Ser), suggesting a mutational hotspot region [51]. In silico predictions as well as several functional studies have confirmed a damaging effect of the variant [52,53,57,58,59,60].

On the contrary, we could not confirm the pathogenicity of another *TRPV4* variant (p.Tyr439Asn) identified in a patient with bilateral clubfoot and lower limb hypotrophy. Although the variant is characterized as damaging using in silico prediction tools with a frequency of less than 0.001% in the gnomAD v2.1.1 dataset and the missense variants in gene *TRPV4* are a common disease-causing mechanism, it is characterized as a variant of uncertain significance (VUS). The unaffected father of the proband was available for testing and targeted direct sequencing of *TRPV4* in the father’s genomic DNA sample confirmed the same heterozygous variant.

The heterozygous likely pathogenic *FLNB* variant (p.Gln685His) was identified in female and male twins with bilateral clubfoot, congenital hip dislocation, congenital knee dislocation, abnormal cartilage morphology, and lower limb hypotrophy. The variant has been previously described in the literature and associated with autosomal dominant Larsen syndrome [61]. Functional studies provided some evidence that the variant may be damaging [61], and in silico predictions support those findings.

Another heterozygous likely pathogenic variant was identified in the splice region of gene *FOXP1* (c.1723-2A>C), in a single patient with clubfoot, cerebral palsy, lower limb hypotrophy, and horizontal nystagmus that was cognitively challenged. This variant has not been reported in other patients, but it is also absent from the population database. Although the segregational analysis is recommended to further support the variant pathogenicity, the patient’s parents were not available for testing. It is predicted that the variant is altering splicing and may disrupt the normal protein structure and function. Additionally, exon skipping is a known disease mechanism in autosomal dominant *FOXP1*-related disorders and multiple pathogenic loss-of-function variants are reported downstream of this variant [62,63,64]. The clinical presentation of FOXP1 syndrome includes a wide range of symptoms, such as neurodevelopmental and syndromic clubfoot, which could be a rare feature of this disorder in our patient [65].

Two patients in our study were carriers of large genomic rearrangements. One boy, with bilateral clubfoot, adenoid hypertrophy, and cryptorchism, was a carrier of a heterozygous duplication in the genomic region 17p12. The minimum size of this duplication is 1.4 Mb and it includes eight genes (*COX10*, *CDRT15*, *HS3ST3B1*, *PMP22*, *TEKT3*, *TVP23C-CDRT4*, *CDRT4*, and *TVP23C*). This duplication is classified as pathogenic because it is observed at a very low frequency in the gnomAD SVs v2.1.1 dataset (0.023%) and patients with similar symptoms and duplications of this region have previously been reported [66]. This duplication includes the gene *PMP22*, associated with autosomal dominant Charcot–Marie–Tooth disease, Type 1A (CMT1A). After the proband’s genetic diagnosis, more detailed phenotyping in other family members was performed and CMT1A suspected in the sister and mother. The gene *PMP22* duplication was confirmed in additional family members (mother and sister).

Another heterozygous duplication of approximately 0.5 Mb at the Xq28 genomic region was found in a 4-year-old girl with bilateral clubfoot, lower limb hypotrophy, and overweight. This duplication spans across eight genes (*F8*, *FUNDC2*, *CMC4*, *MTCP1*, *BRCC3*, *VBP1*, *RAB39B*, and *CLIC2*) and is associated with Chromosome Xq28 duplication syndrome. This duplication was reported in the gnomAD SVs v2.1.1 dataset with a very low frequency (0.006%) and several similarly affected patients have been reported with likely pathogenic CNVs in this region [67,68].

There are several limitations to this study. The first limitation refers to the investigation that was performed on a single population (Serbian population); therefore, additional studies should be conducted on different populations. The second limitation refers to the study sample; thus, larger samples should be included in future investigations. Moreover, the heterogeneity of the cohort as well as a high variety of genetic results could be some of the potential limitations in the achievement of significant differences in more clinical and demographic parameters, rather than just in family history.

However, we still believe that our data are valuable in highlighting the genetic heterogeneity of clubfoot etiology and providing insights into different molecular pathways. Furthermore, as more genetically conditioned patients are reported in different populations, more clinically relevant data will be available and WES analysis will become even more accurate and informative in diagnosing genetic causes of clubfoot.

Future research should be focused on identifying specific genes that might be involved in the etiopathogenesis of clubfoot, as well as their role along with interaction with other factors in the expression of such deformity. Furthermore, the evaluation of the influence of specific risk factors, both maternal and fetal, and their interactions during the development of the fetus should also be investigated.

## 5. Conclusions

The results from this study demonstrated that clubfoot family history was significantly associated with the presence of genetic mutations in offspring. From the study group, 14% of the tested subjects were carriers of six different heterozygous variants. All patients with genetic variants had some type of neuromuscular disorder.

Our findings will have an impact on better understanding the role of genetic inheritance on clubfoot etiology and contribute to the establishment of genetic diagnostic strategies in pediatric patients with this condition which can lead to more efficient genetic diagnosis. However, a small sample size, one study population, and the heterogeneity of the cohort should be taken as potential weaknesses in the results’ interpretations.

## Figures and Tables

**Table 1:** Distribution of tested parameters in the study sample.

Variables		Total N = 50	Positive (with Genetic Variant/s) N = 7	Negative (without Genetic Variant/s) N = 43	*p* * Positive/Negative
Gender, N (%)	Male	31 (62%)	4 (57.14%)	27 (62.79%)	1.000
	Female	19 (38%)	3 (42.86%)	16 (37.21%)	
Age, (MV ± SD)		8.82 ± 4.27	5.14 ± 1.68	9.42 ± 4.27	
Dominant foot, N (%)	Right	33 (66%)	7 (100%)	26 (60.47%)	0.080
	Left	17 (34%)	0 (0)	17 (39.53%)	
Affected foot, N (%)	One foot	13 (26%)	0 (0)	13 (30.23%)	0.168
	Both feet	37 (74%)	7 (100%)	30 (69.77%)	
Deformity degree, N (%)	I	9 (18%)	0 (0)	9 (20.93%)	0.270
	II	17 (34%)	3 (42.86%)	14 (32.56%)	
	III	14 (28%)	1 (14.29%)	13 (30.23%)	
	IV	10 (20%)	3 (42.86%)	7 (16.28%)	
Treatment, N (%)	Ponseti method	14 (28%)	1 (14.29%)	13 (30.23%)	0.657
	Ponseti method and surgery	36 (72%)	6 (85.71%)	30 (69.77%)	
Neuromuscular disorders, N (%)	Yes	21 (42%)	5 (71.43%)	16 (37.21%)	0.115
	No	29 (58%)	2 (28.57%)	27 (62.79%)	
Clubfoot family history, N (%)	Positive	10 (20%)	4 (57.14%)	6 (13.95%)	0.023
	Negative	40 (80%)	3 (42.86%)	37 (86.05%)	
Maternal smoking, N (%)	Yes	16 (32%)	1 (14.29%)	15 (34.88%)	0.406
	No	34 (68%)	6 (85.71%)	28 (65.12%)	

Positive—group with genetic variant/s; Negative—group without genetic variant/s; * Fisher’s test; MV—mean value; SD—standard deviation.

**Table 2:** Individual characteristics of genetically positive patients in the study sample.

Variables	Patient 1	Patient 2	Patient 3	Patient 4	Patient 5	Patient 6	Patient 7
Gender	Male	Male	Male	Female	Male	Male	Female
Age * (years)	8	4	6	6	3	5	4
Dominant foot	Right	Right	Right	Right	Right	Left	Right
Affected foot	Both feet	Both feet	Both feet	Both feet	Both feet	Both feet	Both feet
Degree	II	III	II	III	III	II	III
Treatment	Ponseti method and Surgery	Ponseti method	Ponseti method and Surgery	Ponseti method and Surgery	Ponseti method and Surgery	Ponseti method and Surgery	Ponseti method and Surgery
Neuromuscular disorders	Charcot–Marie–Tooth type Ia	Hereditary motor and sensory neuropathy type IIc	FOXP1-related disorder	Larsen syndrome	Larsen syndrome	TPRV4-related disorder	Xq28 duplication syndrome
Clubfoot family history	Positive	Negative	Negative	Positive	Positive	Negative	Positive
Maternal smoking	No	No	No	No	No	No	Yes

*—on examination.

**Table 3:** Characterization of identified variants.

Patient ID	P19	P31	P33	P37	P38	P40	P49
Gene	*COX10*, *CDRT15*, *HS3ST3B1*, *PMP22*, *TEKT3*, *TVP23C-CDRT4*, *CDRT4*, *TVP23C*	*TRPV4*	*FOXP1*	*FLNB*		*TRPV4*	*F8*, *FUNDC2*, *CMC4*, *MTCP1*, *BRCC3*, *VBP1*, *RAB39B*, *CLIC2*
Gene Transcript	NC_000017.10	NM_021625.5	NM_001349338.3	NM_001457.4		NM_021625.5	NC_000023.10
Nucleotide Change	g.(?_14095306)_ (15466797_?)dup	c.806G>A	c.1723-2A>C	c.2055G>C		c.1315T>A	g.(?_154128141)_ (154563736_?)dup
Variant type	Duplication	Missense	Splice	Missense		Missense	Duplication
Zygosity	Heterozygous	Heterozygous	Heterozygous	Heterozygous		Heterozygous	Heterozygous
Protein Transcript	NA	NP_067638.3	NP_001336267.1	NP_001448.2		NP_067638.3	NA
Amino Acid Change	NA	p.Arg269His	NA	p.Gln685His		p.Tyr439Asn	NA
	Pathogenic	Pathogenic	Likely pathogenic	Likely pathogenic		VUS	Pathogenic
Population Data (%) ^1^	0.023	0	0	0		<0.001	0.006
Disorder	Charcot–Marie–Tooth disease, Type 1A	Hereditary motor and sensory neuropathy, Type IIc	FOXP1-related disorder	Larsen syndrome		TRPV4-related disorder	Chromosome Xq28 duplication syndrome
OMIM ID	118220	606071	605515	150250		605427	300815
Mode of inheritance	Autosomal dominant	Autosomal dominant	Autosomal dominant	Autosomal dominant		Autosomal dominant	X-linked dominant

^1^ gnomAD v2.1.1 dataset.

## Data Availability

The data presented in this study are available on reasonable request from the corresponding author. The data are not publicly available due to privacy restriction.
